# Advancements in computational modelling of biological systems: seventh annual SysMod meeting

**DOI:** 10.1093/bioinformatics/btad539

**Published:** 2023-09-14

**Authors:** Bhanwar Lal Puniya, Andreas Dräger

**Affiliations:** Department of Biochemistry, University of Nebraska-Lincoln, Lincoln, NE 68588, United States; Computational Systems Biology of Infections and Antimicrobial-Resistant Pathogens, Institute for Bioinformatics and Medical Informatics (IBMI), Eberhard Karl University of Tübingen, Tübingen 72076, Germany; Department of Computer Science, Eberhard Karl University of Tübingen, Tübingen 72076, Germany; German Center for Infection Research (DZIF), Partner Site Tübingen, Tübingen 72076, Germany; Cluster of Excellence ‘Controlling Microbes to Fight Infections’, Eberhard Karl University of Tübingen, Tübingen 72076, Germany

## Abstract

**Summary:**

The Computational Modelling of Systems Biology (SysMod) Community of Special Interest (COSI) convenes annually at the Intelligent Systems for Molecular Biology (ISMB) conference to facilitate knowledge dissemination and exchange of research findings on systems modelling from interdisciplinary domains. The SysMod meeting 2022 was held in a hybrid mode in Madison, Wisconsin, spanning a 1-day duration centred on modelling techniques, applications, and single-cell technology implementations. The meeting showcased innovative approaches to modelling biological systems using cell-specific and multiscale modelling, multiomics data integration, and novel tools to develop systems models using single-cell and multiomics technology. The meeting also recognized outstanding research by awarding the three best posters. This report summarizes the key highlights and outcomes of the meeting.

**Availability and implementation:**

All resources and further information are freely accessible at https://sysmod.info.

## 1 Introduction

The SysMod community operates as a forum within the International Society for Computational Biology (ISCB), serving as an essential platform for exchanging ideas and discussing advancements in systems biology modelling and bioinformatics techniques (Dräger *et al.* 2021). The community aims to comprehend the complexities of biological systems and their applications in health, medicine, agriculture, and industry. To achieve this goal, the SysMod community organizes an annual meeting to facilitate discussions and collaborations among researchers, academics, and industry experts. The seventh edition of this annual meeting was held during the Systems for Molecular Biology (ISMB) conference in 2022, featuring a hybrid mode of participation that accommodated both virtual and in-person attendance. The participants from around the world had the opportunity to engage in vibrant discussions and share insights on the latest research and developments in the field of systems biology.

## 2 The event

### 2.1 Session I: methods for modelling and applications

Dr Andreas Dräger moderated the first session and delivered the welcome speech. The session featured a keynote talk from Dr Reinhard Laubenbacher on ‘Multiscale computational models for lung immunity’. The talk highlighted the development of a complex and hybrid model to investigate how the host fights respiratory fungal infection invasive aspergillosis (Ribeiro *et al.* 2022) and its application in investigating the potential mechanism responsible for pulmonary aspergillosis in immunocompetent COVID patients. Next, Dr Patrice Ravel presented an approach integrating Modular Response Analysis with multiple linear regression (Borg *et al.* 2023) for biological network inference, showing applications for two networks, including a six-module MAP kinase network. The following talk was delivered by Dr Peter Karp and focused on visualizing organism-scale metabolic networks based on pathway tools (Paley *et al.* 2021). He demonstrated the creation of organism-specific, searchable, and real-time zoomable metabolic charts. The next talk was from Dr Nathan E. Lewis, who presented investigations in human milk oligosaccharide (HMO) biosynthesis through metabolic modelling with multiomics integration (Kellman *et al.* 2022). His work identified biosynthetic pathways for HMOs and the best models and candidate genes for glycosyltransferase. The first session ended with two flash talks. Lavanya Raajaraam discussed the Co-FSEOF framework to study metabolite coproduction (Raajaraam and Raman 2021) and Arda Halu presented MuXTalk, a tool to model signalling crosstalk using multilayer networks (Halu *et al.* 2022).

### 2.2 Session II: modelling signal transduction, gene regulation, and protein–protein interactions

Dr Bhanwar Lal Puniya moderated the second session. He began with a talk by Dr Nathan P Manes on the data-driven computational modelling of toll-like receptor signalling of mouse macrophages to facilitate simulation at the level of molecular interactions. The subsequent presentation was delivered by Aurelien Pelisiser, who discussed modelling cell-type-specific gene regulation in rheumatoid arthritis. He identified key genes driving rheumatoid arthritis and inferred a gene regulatory network in synovial tissue. The session’s final presentation was delivered by Eric Bell, who presented a program suite comprising three tools, including the PEPPI pipeline, for protein-protein interaction network analysis, applied to SARS-CoV2 and human interactome to predict cross-species interactions (Bell *et al.* 2022).

### 2.3 Session III: applications of single-cell technology

The final session was moderated by Dr Andreas Dräger and opened with a talk from Kang Jin on the inference of perturbation responses in temporally sampled single-cell data using the CellDrift framework (Jin *et al.* 2022). The next talk was from Qian Qin on the Pyro-Velocity, a framework that infers RNA velocity from single-cell (Qin *et al.* 2022). In the third talk, Shaimaa Bakr presented SparseGMM, a module network approach for gene regulatory network inference (Bakr *et al.* 2023), which was applied to healthy liver tissue and liver cancer samples to identify robust modules. Dr Joseph Wayman delivered the following presentation on building gene regulatory networks of Tfh10 cells using single-cell genomics to understand immune responses during ageing (Wayman *et al.* 2023). Prof. Ana Conesa delivered the second keynote talk on ‘The integration of multiomics data to infer multilayered systems biology models’. She discussed challenges and methods for multiomics integration and presented various applications of multilayered models. These include the MORE algorithm to explain gene expression and identify potential regulators and multiomics flux balance analysis to understand the link between epigenetics and metabolism (Ugidos *et al.* 2022).

### 2.4 Poster session

SysMod 2022 meeting featured 29 posters and selected the three best posters (Table 1), which Dr Bhanwar Lal Puniya announced at the end of the meeting.

**Table 1.** Poster awards.

Prize	Winner	Title
First	Carolina I Larkin	A mechanistic model of alphavirus replication within a mammalian host cell
Second	Kang Jin	CellDrift: inferring perturbation responses in temporally sampled single cell data
Third	Shaimaa Bakr	Identifying key multifunctional components shared by critical cancer and normal liver pathways via sparseGMM

## 3 Conclusion

The seventh Annual SysMod meeting showcased cutting-edge research and developments in systems biology, bioinformatics, and their applications across various biological systems. It provided a platform for experts to discuss advances in modelling methodologies, biological networks, and single-cell technology applications. The SysMod 2022 meeting enabled knowledge exchange, furthering the community’s efforts to understand the complexities of biological systems and their applications in medicine, agriculture, and industry.

## Data Availability

For further general and up-to-date information about SysMod, we refer the reader to https://sysmod.info.
